# Necroptosis, pyroptosis and apoptosis: an intricate game of cell death

**DOI:** 10.1038/s41423-020-00630-3

**Published:** 2021-03-30

**Authors:** Damien Bertheloot, Eicke Latz, Bernardo S. Franklin

**Affiliations:** 1 Institute of Innate Immunity, University Hospitals Bonn, University of Bonn, Bonn, NRW Germany; 2 Department of Infectious Diseases and Immunology, University of Massachusetts Medical School, Worcester, MA USA; 3 German Center for Neurodegenerative Diseases, Bonn, NRW Germany

**Keywords:** Apoptosis, Necroptosis, Pyroptosis, Inflammation, Survival, Immune cell death, Innate immunity

## Abstract

Cell death is a fundamental physiological process in all living organisms. Its roles extend from embryonic development, organ maintenance, and aging to the coordination of immune responses and autoimmunity. In recent years, our understanding of the mechanisms orchestrating cellular death and its consequences on immunity and homeostasis has increased substantially. Different modalities of what has become known as ‘programmed cell death’ have been described, and some key players in these processes have been identified. We have learned more about the intricacies that fine tune the activity of common players and ultimately shape the different types of cell death. These studies have highlighted the complex mechanisms tipping the balance between different cell fates. Here, we summarize the latest discoveries in the three most well understood modalities of cell death, namely, apoptosis, necroptosis, and pyroptosis, highlighting common and unique pathways and their effect on the surrounding cells and the organism as a whole.

## Introduction

Cell death has physiological and pathological functions. The cellular lifespan can range from a few days to many years, depending on the cell type. Many physiological processes require cell death for their function (e.g., embryonal development and immune selection of B and T cells). Hence, each day, billions of cells die and are quickly removed by phagocytes. This mechanism of dead cell removal/clearance operates smoothly under normal conditions, demonstrating the efficacy of phagocytic processes. However, this system can be overwhelmed when large numbers of cells die abruptly and accumulate, such as during infection, chronic inflammation, and tissue damage. Sudden and unrestrained cell death results in massive release of cellular contents into the extracellular space. Released molecules act as damage signals, known as danger-associated molecular patterns (DAMPs). The presence of DAMPs in the extracellular space elicits a robust immune response that recruits additional phagocytes and other immune cells to clear the threat and promote tissue repair. During infections, the presence of pathogen-associated molecular patterns (PAMPs) elicits specific antimicrobial immune responses to control the infection. In certain circumstances, cells can regulate (or “program”) their death to tailor immune responses, thereby changing the impact their death will have on the surroundings. The best-studied forms of programmed cell death are apoptosis, necroptosis, and pyroptosis. Additional types of programmed cell death that occur in particular cell toxicity states often induced by pharmacological treatments (e.g., anticancer therapies) have been reported.^1^ Whether these less-studied pathways have physiological functions and how they diverge from other better understood pathways requires further investigation. Of note, a nomenclature to distinguish the many and often intercrossed cell death pathways has been proposed.^2^ A dichotomy has long dominated the general understanding of how dying cells affect their surroundings: necrosis, regarded as a passive and highly inflammatory form of cell death, and apoptosis, considered to be highly coordinated and immunologically inert (or “silent”). While necroptosis and pyroptosis act as “whistle blowers”, resulting in the release of alarmins and other proinflammatory signals into the cellular surroundings, apoptosis is considered “silent” and dampens subsequent immune responses.

Although this simplistic view has blurred the intricate mechanisms separating these forms of cell death, recent studies have uncovered new effectors and cell death pathways and revealed a more complex and intertwined landscape of what we call cell death. Here, we review the current understanding of programmed cell death. We focus on apoptosis, necroptosis, and pyroptosis and on how these processes regulate the immune response.

## Necroptosis: regulated necrosis with passive and active proinflammatory functions

### Mechanisms of action and regulation of necroptosis

Apoptosis was long thought to be the only regulated cell death pathway. In addition, its counterpart necrosis was considered to be rather ‘clumsy’ and to culminate with the loss of membrane integrity and passive leakage of intracellular contents. It has now become clear that a nonapoptotic form of cell death exists that has evolved to detect pathogens and promote tissue repair. This type of regulated cell death, coined necroptosis, occurs following the activation of the tumor necrosis receptor (TNFR1) by TNFα,^3^ even though TNFα has long been considered an inducer of apoptosis.

**Fig. 1:**
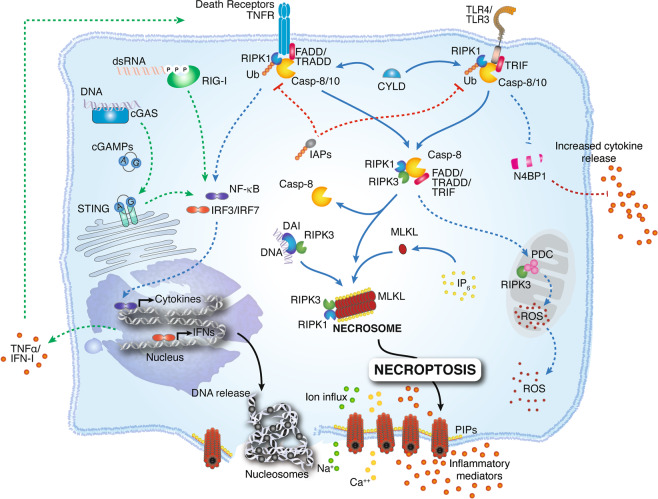
Necroptosis is triggered downstream of death domain receptors (e.g., TNFR and Fas) and Toll-like receptor (TLR)-4 or TLR3. Upon activation, these receptors recruit the adapter proteins FADD, TRADD, and TRIF, which interact with RIPK1 and caspase-8 or -10. First, RIPK1 is ubiquitylated by IAPs, keeping it nonfunctional and enabling proinflammatory downstream activity via NFκB. After detection of a “death signal”, RIPK1 is deubiquitylated by CYLD and can thus recruit RIPK3. The RIPK1/RIP3 complex recruits and phosphorylates MLKL. In the presence of highly phosphorylated inositol phosphate (IP_6_), phosphorylated MLKL oligomerizes, thus forming the necrosome. MLKL oligomers translocate to phosphatidylinositol phosphate (PIP)-rich patches in the plasma membrane and form large pores. Ultimately, MLKL pores lead to necroptotic cell death by allowing ion influx, cell swelling, and membrane lysis followed by the uncontrollable release of intracellular material. The cytosolic nucleic acid sensors RIG-I and cGAS/STING also contribute to necroptotic cell death, as they induce IFN-I and TNFα and thus promote necroptosis via an autocrine feedback loop. Downstream of TNFR or TLR engagement, active caspase-8 cleaves the cytokine blocker N4BP1, thus promoting an increase in cytokine release. Once activated, RIPK3 phosphorylates the pyruvate dehydrogenase complex (PDC) in mitochondria and promotes aerobic respiration and mitochondrial ROS production. In the presence of cytosolic DNA released from infecting microbes, DNA-dependent activator of IFN regulatory factor (DAI) recruits RIPK3 and thus bypasses RIPK1 for activation of MLKL and formation of the necrosome complex

Activation of other cellular receptors triggers necroptosis. These receptors include death receptors (i.e., Fas/FasL),^4^ Toll-like receptors (TLR4 and TLR3)^5–7^ and cytosolic nucleic acid sensors such as RIG-I and STING, which induce type I interferon (IFN-I) and TNFα production and thus promote necroptosis in an autocrine feedback loop.^8–10^ Most of these pathways trigger NFκB-dependent proinflammatory and prosurvival signals. However, additional inhibition of the proteolytic enzyme Caspase-8 by microbes or pharmacological agents triggers the necroptotic pathway (Fig. 1). Downstream of the abovementioned receptors, active RIPK1 is recruited within an oligomeric complex that includes FADD, caspase-8 and caspase-10.^11,12^ In the absence of caspase-8 activity, RIPK1 recruits and phosphorylates RIPK3, forming a complex called the ripoptosome.^13,14^ The RIPK1/RIPK3 complex recruits and phosphorylates MLKL, thus forming the necrosome.^15,16^ MLKL phosphorylation induces a conformational change leading to the exposure of a 4-helical bundle domain (4HBD). Initial studies speculated that the RIPK1/RIPK3/MLKL pathway induces cell death through mitochondrial destabilization. This effect occurs via a phosphoglycerate mutase family member 5 (PGAM5)- and dynamin-related protein 1 (DRP1)-dependent pathway.^17^ However, mice with genetic deficiency of *Pgam5* or *Drp1* display no alterations in TNF-induced necroptosis, challenging this theory.^16,18^ Further disproving the potential involvement of mitochondria in necroptosis induction, the use of the ROS scavenger butylated hydroxyanisole did not affect TNF-induced necroptosis.^19^ Instead, two nonexclusive models explain how MLKL compromises cellular integrity: (1) MLKL constitutes a platform at the plasma membrane for the opening of calcium or sodium ion channels, thus enabling ion influx, cell swelling, and rupture,^20,21^ and (2) MLKL itself forms pores in the plasma membrane through interaction between a positively charged patch in the 4HBD and negatively charged phosphatidylinositol phosphates (PIPs) present at the membrane.^22–24^ In addition, MLKL oligomerization and membrane translocation seem to depend on a specific inositol phosphate (IP) code.^25^ Indeed, Dovey and colleagues demonstrated that phosphorylation of MLKL by RIPK3 alone is not sufficient for MLKL translocation to the membrane. Instead, MLKL requires the interaction of its N-terminal domain with highly phosphorylated IPs (e.g., IP_6_). This interaction, in turn, displaces the sixth α-helix of MLKL, which acts as a molecular brace believed to inhibit interactions with the MLKL N-terminal domain and control MLKL oligomerization.^24^ In line with these results, expression of the MLKL^D139V^ mutant, which alters the two-helix brace structure, endows MLKL with RIPK3-independent constitutive killing activity, causing lethal postnatal inflammation in homozygous mutant mice.^26^ Moreover, MLKL oligomerization was recently shown to dictate the kinetics and threshold of necroptotic cell death. Indeed, phosphorylated MLKL first assembles into cytosolic polymeric necrosomes and then traffics with tight junction proteins to the plasma membrane, where both accumulate to form micron-sized structures.^27^ Although mitochondrial damage and ROS production are not considered to be directly involved in the establishment of necroptotic cell death, a recent study by Yang and colleagues showed that RIPK3 instead has downstream effects on mitochondria: RIPK3 directly phosphorylates and activates the E3 subunit of the pyruvate dehydrogenase complex and promotes aerobic respiration and mitochondrial ROS production.^28^ This finding could explain the link between necroptosis and mitochondrial destabilization.

Given its powerful and nonreversible nature, the necroptotic pathway’s early steps must be heavily regulated. Indeed, upon TNFR1 engagement, RIPK1 is rapidly recruited to signaling complex I, where it interacts with TRADD and TRAF2. At this location, TRAF2 and TRAF5 control the polyubiquitylation of RIPK1 via cIAP1/2, limiting the cell death function of RIPK1.^29–34^ Similarly, after TLR activation (e.g., by LPS or poly-(I:C)), the function of RIPK1/3 is regulated by cIAP1/2 and XIAP through ubiquitylation.^30,31^ Importantly, ubiquitylation of RIPK1 and RIPK3 not only prevents cell death but also is essential for NFκB-dependent induction of proinflammatory genes.^35^ Furthermore, low extracellular pH was recently shown to act on a highly conserved histidine (His^151^) in the amino acid sequence of RIPK1, thus inhibiting its kinase activity and preventing cell death.^36^ In addition to these in vitro data, generating knockin mice expressing *Ripk1*^*H151N*^ would undoubtedly help determine the physiological function of this pH sensitivity of RIPK1.

Contributing to the “death signal”, CYLD deubiquitylates TRAF2 and RIPK1, allowing the formation of the ripoptosome.^37–41^ The mitochondrial protein Smac further promotes necroptosis by triggering proteasomal degradation of cIAP1/2 and XIAP.^42^ Consequently, Smac mimetics have been developed as therapeutic tools against cancer cells or HIV-infected cells.^43–45^ These agents and other anticancer drugs promote the formation of the ripoptosome through depletion of XIAP and cIAP1/2 independent of death receptor activation.^11^

The immune system has evolved a way to bypass inhibition upstream of RIPK3 as a rapid countermeasure to block viral spread. Indeed, the murine cytomegalovirus (MCMV) DNA genome was found to activate the DNA-dependent activator of IFN regulatory factor (DAI or ZBP1), which binds directly to RIPK3 through its RHIM domain, virtually bypassing RIPK1.^46,47^ More recently, Lim et al. proposed a more ambivalent role for DAI in regulating necroptosis in macrophages.^7^ These authors demonstrated that despite driving necroptosis in the absence of RIPK1, DAI protects autophagy-incompetent *Atg16l1*^−/−^ cells from necroptosis. Thus, depending on the context, DAI acts as either an activator or a suppressor of necroptosis. Since inhibition of autophagy results in the accumulation of DAI,^48^ it can be speculated that a certain threshold concentration of DAI is necessary for it to execute its prosurvival function. However, precisely how these opposite functions of DAI are regulated remains to be elucidated.

Notably, DAI is not the only RHIM domain-containing protein capable of activating RIPK3 in the absence of RIPK1. Indeed, TRIF is also known to interact with and directly activate RIPK3 in the absence of RIPK1.^6^ Remarkably, during embryonic development, RIPK1 plays a crucial prosurvival role that is independent of its kinase activity.^49–51^ Indeed, *Ripk1*^−/−^ mice die soon after birth due to uncontrolled caspase-8- or RIPK3-dependent apoptosis or necroptosis, respectively. Presumably, RIPK1-RHIM usually acts as a “sponge” preventing the activation of RIPK3/MLKL signaling by TRIF or DAI, pathways that are currently identified as effectors of the lethality of *Ripk1* knockout.^52^ Collectively, these data demonstrate the essential role of the RHIM domain interaction as both a trigger and a regulator of necroptosis. The RHIM-containing protein that is responsible for pressing the “RIPK3 trigger” seems to depend on the cellular context and the upstream pathway activated. A more extensive study of the regulatory processes involved in the induction of necroptosis is necessary to fully solve this puzzle. Further complicating the understanding of necroptosis’s regulatory mechanism, mouse and human MLKL orthologs have species-specific requirements for interaction with their cognate RIPK3 partners due to the coevolution of both the MLKL C-terminal pseudokinase domain and the RIPK3 kinase domain.^53^ Hence, although the mechanism of MLKL-dependent plasma membrane permeabilization is similar, direct transposition of regulatory mechanisms between species may be too simplistic and should be proposed cautiously.

### Necroptosis in infection and sterile inflammation

Necroptosis has mainly been studied downstream of the activation of death domain receptors or pattern recognition receptors (PRRs) as one response to microbial infection. For example, in the context of *Yersinia* infection, RIPK3-mediated necroptosis is beneficial to the host.^54,55^ On the other hand, lung infection with *Staphylococcus aureus* induces necroptotic epithelial cell death, which is detrimental to the host.^56^ Interestingly, recent work indicates that microbes can directly target the necroptotic pathway to prevent cell death and promote their replication. Indeed, the enterohemorrhagic *Escherichia coli* (EHEC)-expressed virulence effector NleB1 modifies arginine residues in TRADD, FADD, and RIPK1, which blocks apoptosis and necroptosis.^57,58^ Some viruses have also evolved immune evasion strategies to prevent cell death: the MCMV-encoded viral inhibitor of RIP activation (vIRA) directly targets the DAI-RIPK3 complex through its own RHIM domain, thus preventing necroptosis and allowing viral replication.^46,59^ Of note, vIRA was shown to prevent necroptosis downstream of Fas and TNFR1^60,61^ or TLR3 and TLR4^6,60^ by targeting RIPK1/RIPK3 or TRIF/RIPK3, respectively. Although MCMV-encoded vIRA induces NFκB early during infection, at later time points, it blocks NFκB-dependent cytokine induction and proinflammatory signaling downstream of TNFR1 by targeting RIPK1 and NEMO for proteasomal or lysosomal degradation, thus covering all loose ends.^60,62,63^ Similarly, HCMV has developed countermeasures to prevent necroptosis, but in contrast to those of MCMV, these actions of HCMV seem to happen downstream of RIPK3/MLKL activation.^64^ A recent study by Fletcher-Etherington et al. proposed HCMV-encoded vICA as an inhibitor of necroptosis through direct interaction with MLKL.^65^ In humans, herpes simplex virus-1 and -2 also encode the RHIM-containing proteins ICP6 and ICP10, which block TNF-induced necroptosis by preventing the interaction of RIPK1 and RIPK3 through a RHIM-mediated decoy.^66^ Interestingly, in mice, ICP6 has the opposite effect, as it induces necroptosis through direct interaction with RIPK1 and RIPK3,^67^ further underscoring the differences between mouse and human necroptotic mechanisms.

The fact that viruses have evolved tools to escape necroptotic cell death is a testimony to this pathway’s importance in fighting microbial infection. Once activated, necroptosis triggers a process of cellular autodestruction, resulting in passive release of cytokines, DAMPs, and PAMPs. These mediators provide proinflammatory cues that recruit immune cells to stimulate efficient antimicrobial responses and activate the tissue repair machinery. Upon cellular erosion, elements of the infecting microbe are released into the extracellular environment, where they activate well-known proinflammatory pathways through a large variety of PRRs.

In sterile inflammation, such as during the onset of a particular cancer, necroptosis is essential for the immune system to mount an adequate response. Low MLKL expression has been associated with poor prognosis in patients with breast, ovarian, gastric, colon, and pancreatic cancers.^68^ In animal studies, intratumoral delivery of an autoactive RIPK3 construct or the use of necroptotic cells as a vaccination strategy generated efficient antitumoral immune responses.^69,70^ Interestingly, effective antitumor immunity depends on the NFκB pathway rather than on MLKL-dependent disruption of cell integrity. This pattern indicates that cytokine release or other events (e.g., posttranslational modification of effector proteins) triggered by the NFκB pathway are important messengers released from dying cells. For instance, proper cross-priming of effector CD8^+^ T cells requires the RIPK1/RIPK3 pathway and NFκB signaling^71^ and is responsible for the antitumor immune response induced by necroptotic cell death.^70^ Hence, necroptosis is an essential proinflammatory cell death pathway not only because of the uncontrolled release of cellular content resulting from membrane permeabilization but also because of the well-regulated proinflammatory pathways triggered in parallel to necrosome assembly, which allow the expression of proinflammatory cytokines before cell disintegration.

Further demonstrating that there is more to necroptosis than passive release of cellular content, recent work by Tanzer et al. proposed the existence of a kinetically regulated necroptotic secretome.^72^ Using unbiased label-free mass spectrometry, the authors found that at early time points (3 h) following activation of necroptosis, myeloid cells boost the release of conventionally secreted proteins through the ER-Golgi apparatus. Moreover, dying cells also amplify the production of extracellular vesicles enriched with MLKL, a previously described necroptosis-counteracting mechanism.^73,74^ However, at later time points (5–7 h), the levels of cytokines usually released through the conventional secretory pathway (e.g., CCL2 and IL-8) are reduced in the secretome of necroptotic cells. Instead, late-stage necroptotic cells use lysosomal exocytosis for the release of intracellular content.

Interestingly, exocytosis has already been described as a membrane repair mechanism triggered upon membrane permeabilization and as an ancient mechanism to remove infecting microbes.^75–78^ It is tempting to speculate that these processes of early shedding of MLKL from intact cells followed by lysosomal exocytosis-mediated membrane repair exist to buy more time for the cells to increase cytokine production and tailor an appropriate proinflammatory signal before cell integrity is lost. This theory fits with the finding that translation from mRNA coding for cytokines continues in necroptotic cells (triggered by TNF or viral infection) even after permeabilization of the plasma membrane to promote the recruitment of immune cells.^79^ In line with these findings, MLKL and RIPK3 levels in ICU patients’ peripheral blood showed little difference from those in peripheral blood from healthy donors upon arrival. However, MLKL and RIPK3 levels increase over time and are predictive of poor patient survival.^80,81^ Interestingly, RIPK3 plasma levels were significantly elevated in ICU patients needing ventilation, leading to ventilator-induced lung injury.^82^ It is worth noting that in the context of COVID-19, critically ill patients require ventilation, which, in some cases, is thought to induce ventilator-induced lung injury. A small study found that the plasma RIPK3 level was positively correlated with the severity of COVID-19 in the patients tested (16 patients total).^83^ These early findings warrant further investigation of a potential role for necroptosis in COVID-19 development and other mechanical ventilation-induced lung injuries. However, one must also point out that none of the mentioned studies used analysis methods specific for active MLKL or RIPK3 (i.e., phosphorylation-specific ELISA). Such tools are now available^84^ and will be required to conclusively determine the role of necroptosis in the development of acute or chronic inflammatory diseases via analysis of patient samples.

Nevertheless, since necroptosis plays a considerable role in the induction of inflammation, it is not surprising that necroptosis is involved in the onset of disease. Several animal studies have demonstrated a role for necroptosis in tissue damage induced by ischemia-reperfusion injury such as that occurring with stroke,^85^ kidney ischemia,^86^ myocardial infarction,^87,88^ or organ transplantation.^89^ Furthermore, RIPK1 and RIPK3 have been implicated in the development of chronic inflammatory diseases such as chronic obstructive pulmonary disease,^90,91^ rheumatoid arthritis,^92^ multiple sclerosis,^93^ and Crohn’s disease^94^ and in the exacerbation of TNF-induced systemic inflammatory response syndrome.^88,95–98^ However, since RIPK1 and RIPK3 have necroptosis-independent functions, it is difficult to decipher the proper role of necroptosis in models using pathway inhibition (e.g., using the RIPK1 inhibitor Nec-1) or *Ripk1/Ripk3* gene deletion approaches. Some studies have already shown a role for RIPK3 in inducing MLKL-independent inflammation.^92^ Using tools such as knockin mice expressing mutants of RIPK1 or RIPK3 with specific domain alterations inhibiting only part of their function may help to separate the necroptotic effects from the NFκB-related proinflammatory functions of these multitasking proteins.

## Pyroptosis: a master regulator of inflammation

Pyroptosis is a type of cell death culminating in the loss of plasma membrane integrity and induced by activation of so-called inflammasome sensors. These include the Nod-like receptor (NLR) family, the DNA receptor Absent in Melanoma 2 (AIM2) and the Pyrin receptor. Inflammasome sensors detect a variety of PAMPs and DAMPs released by infecting microbes or through dysregulated cellular pathways. Thus, inflammasomes constitute a strong defense against pathogens or cell stress, which results in lytic cell death, preventing microbial spread, while alerting the immune system of imminent danger. However, unbalanced activation of this essential physiological sentinel function leads to the development of pathological inflammation. NLRP3 is the inflammasome sensor most strongly associated with the development of uncontrolled inflammation. For instance, recent evidence points to a correlation between COVID-19 severity and NLRP3 activation by SARS-CoV-2 infection.^99^ Whether the activation of NLRP3 in these settings relates to its previously described sensing of viral RNA^100,101^ or cellular perturbations remains to be investigated. NLRP3 has also long been studied for its role in chronic inflammatory diseases such as Alzheimer’s disease^102–105^ and atherosclerosis,^106^ with a noticeable impact on health systems worldwide. We recently showed that prolonged intake of a Western diet triggers NLRP3-dependent epigenetic reprogramming of granulocyte/monocyte precursors in bone marrow.^107^ Interestingly, the NLRP3 systemic inflammatory signature induced by the Western diet remained imprinted in myeloid cells even after a change to a healthier diet. This is an excellent example of the extent to which pyroptotic cell death affects organisms and explains how crucial physiological control mechanisms are to avoiding undesired inflammation.

### Mechanisms of inflammasome activation

**Fig. 2:**
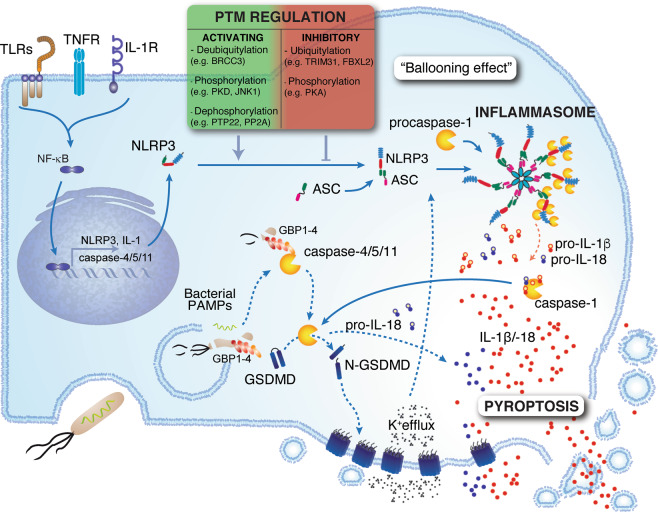
Pyroptosis is an inflammatory form of cell death triggered by intracellular sensors such as NLRP3 (used here as an example) that detect DAMPs, PAMPs, membrane disturbances, osmotic imbalances, and ion efflux, among other stimuli. Upon activation, these sensors recruit the adapter ASC, forming a micron-sized structure called the inflammasome. These oligomeric structures act as platforms for the activation of caspase-1. Active caspase-1 cleaves proforms of the interleukin-1 family cytokines IL-1β and IL-18. Caspase-1 also activates gasdermin D (GSDMD), unmasking the N-terminal domain, which forms pores in the plasma membrane, in turn enabling the release of mature IL-1β and IL-18 and causing cell swelling (a “ballooning effect”) and pyroptosis. In addition, intracellular recognition of LPS can result in pyroptosis: guanylate-binding proteins (GBPs) bind to the surface of bacteria and assemble a caspase-4/11-activating platform composed of GBP1, 2, 3, and 4, and the cytosolic precursors of caspase-4 and -11. Active caspase-4 and -11 cleave GSDMD, leading to pyroptosis. Notably, caspase-4 can additionally induce proteolytic cleavage of IL-18. As an inherently inflammatory form of cell death, pyroptosis has several layers of regulation. Expression of NLRP3 and the cytokine IL-1 requires priming via stimulation of TLRs, TNFR or IL-1R followed by NFκB activation. Inflammasome sensors are heavily regulated through posttranslational modifications, such as phosphorylation and ubiquitylation (indicated in the box) or by cleavage

Mechanistically, when activated, inflammasome sensors oligomerize and recruit the adapter protein apoptosis-associated speck-like protein containing a CARD (ASC), thus seeding the formation of micron-sized polymeric structures known as inflammasomes or “ASC specks” (Fig. 2). Within the canonical pathway, inflammasomes act as a platform for activating the proteolytic enzyme caspase-1, which dimerizes as a proform and generates the fully active p33/p10 species through autoproteolysis.^108^ Active caspase-1 can then process the cytokines interleukin (IL)-1β and IL-18, also expressed as proforms; thus, active caspase-1 is required for the maturation of these respective ILs into 17 and 18 kD active cytokines. Activation of caspase-1 also enables processing of gasdermin D (GSDMD) into a 30 kD fragment able to oligomerize and lock into the plasma membrane, thus forming pores.^109–112^ GSDMD cleavage relies on the presence of its C-terminal domain for recruitment of caspase-1, while the GSDMD N-terminal domain exhibits propyroptotic activity.^113^ In more advanced stages, GSDMD pores lead to membrane destabilization and cell lysis, enabling the release of DAMPs such as HMGB1 that are too large to escape the cell through the pores.^114^ Adding to the complexity of pyroptosis, several additional caspases are implicated in a noncanonical pathway, directly targeting GSDMD for cleavage. Murine caspase-11 was first described as a caspase-1 interactor more than two decades ago.^115^ It was later accepted as an effector of LPS lethality in its own right^116–119^ and was more recently shown to promote pyroptosis in the absence of caspase-1 by directly targeting GSDMD upon stimulation with intracellular LPS.^120,121^ In humans, detection of intracellular LPS is mediated by caspase-4 and -5, which also cleave GSDMD in the absence of caspase-1.^121^ Remarkably, these proinflammatory caspases not only mediate the lethality of intracellular LPS but also act as receptors. Indeed, caspase-4 and -11 interact directly with LPS, which causes their oligomerization and autoactivation.^122^ However, how LPS is detected by caspase-4 and -11 remained elusive until recently, when Santos et al. demonstrated that the activation of TLR4 by LPS initiates an IFN response that triggers the expression of guanylate-binding proteins (GBPs).^123^ GBPs are essential for the activation of caspase-11. In human cells, GBPs are recruited to the *Salmonella* bacterial surface after they escape the vacuole.^124^ GBP1 binds to LPS with high affinity, allowing the formation of a multimolecular complex with GBP2 and GBP4, which then promotes recruitment and activation of caspase-4. Even though caspase-11 and caspase-4 promote pyroptotic cell death, their mechanism differs from that of caspase-1. Indeed, they mediate pyroptosis without enabling maturation of the proinflammatory cytokines IL-1β or IL-18, long considered a signature of pyroptotic cell death. IL-1β and IL-18 still rely on NLRP3 activation by GSDMD-dependent K^+^ efflux and ASC/caspase-1-dependent downstream signaling. These findings highlight the dual role of GSDMD in pyroptosis—not only executing pyroptosis but also promoting its amplification. ASC specks themselves are released from pyroptotic cells and remain stable for several days in tissues or the circulation,^125,126^ thus extending their proinflammatory potential. Consistent with their extracellular proinflammatory potential, extracellular ASC specks accumulate in the brain and act as seeds for amyloid-β and tau protein deposition and plaque formation, hallmarks of neuroinflammation.^102–105^ Hence, the ability of intracellular LPS and GSDMD pores to activate NLRP3 may constitute a mechanism to further extend the scope and duration of the proinflammatory effect of pyroptosis.

### Regulation of pyroptosis

After the activation of inflammasomes, the ESCRT-dependent membrane repair pathway first attempts to contain the damage inflicted by GSDMD pores.^127^ However, if this last attempt to survive is unsuccessful, GSDMD-mediated loss of membrane integrity results in pyroptosis. It is interesting to note that release of IL-1β and IL-18 does not require complete loss of membrane integrity, as both cytokines are released through GSDMD pores in the absence of membrane lysis (i.e., in the presence of glycine).^128,129^ Membrane repair through the ESCRT pathway is thus preferentially a modulator of the immune response downstream of inflammasome activation by allowing cytokine release but limiting—to a certain extent—the uncontrolled release of cellular content. Moreover, in neutrophils, GSDMD pores downstream of NLRC4 activation are uncoupled from cell death but enable IL-1β secretion.^130,131^ This resistance of neutrophils to pyroptosis is possible because of the intracellular localization of GSDMD pores withing membranes of granules and autophagosomes, facilitating sustained release of mature IL-1β while preventing plasma membrane lysis.^131^ Adding to the understanding of GSDMD-independent release of IL-1β, Monteleone et al. used a combination of GSDMD knockout and overexpression of mature IL-1β to demonstrate that caspase-1 cleavage of pro-IL-1β is essential and sufficient for its release from macrophages.^132^ In this model, caspase-1 cleavage of GSDMD merely accelerated the release of IL-1β. They showed that a polybasic motif in mature IL-1β allows its relocation to plasma membrane areas enriched in the phospholipid PIP2. From those locations, active IL-1β may be released via membrane ruffling. Many GSDMD-independent mechanisms have been proposed, including packaging of IL-1β into secretory lysosomes, release in ectosomes or exosomes, and even release in secretory autophagosomes.^132^ The settings in which cells commit to full pyroptosis or restricted IL-1β release are not fully understood. The regulatory mechanisms governing this decision might be cell-specific and influenced by the local requirements and kinetics of IL-1β responses.

Furthermore, GSDMD knockout does not seem to block cell death entirely. In a study by Kayagaki et al., macrophages derived from two different *Gsdmd*^−/−^ mouse strains could not rescue canonical inflammasome-mediated cell death as measured by the release of lactate dehydrogenase.^120^ This inability indicates that pyroptosis encompasses more than simply GSDMD-dependent membrane destabilization. In line with this finding, the group of Andrew Bowie recently described a role for the TLR-IL-1R domain-containing family protein SARM in uncoupling GSDMD activation from IL-1β release and pyroptosis.^133^ They showed that in macrophages, SARM inhibits IL-1β maturation by upstream regulation of NLRP3 activation while promoting mitochondrial depolarization-dependent pyroptosis. The same group previously described a role for SARM as an inhibitor of TLRs.^134^ Whether this newly identified role of SARM is linked to its function as a TLR antagonist (e.g., regulating cell priming upstream of inflammasome activation) remains to be investigated.

Recent advances in understanding the mechanisms modulating events upstream of GSDMD activation and pyroptosis have revealed several posttranslational modifications of NLRP3. NLRP3 can be ubiquitylated and targeted for proteasomal degradation by TRIM31 in peritoneal macrophages.^135^ Interestingly, LPS treatment increases the expression of TRIM31, indicating a potential regulatory feedback mechanism to diminish the inflammatory response. Similarly, in lung epithelial cells, the E3 ubiquitin ligase FBXL2 was proposed as another enzyme capable of preventing NLRP3 activation by targeting it to the proteasome.^136^ Another E3 ubiquitin ligase, MARCH 7, was shown to inhibit NLRP3 downstream of dopamine-induced D1 receptor signaling.^137^ Conversely, after priming by LPS, FBXL2 itself is ubiquitylated by FBXO3 and targeted to the proteasome,^136^ releasing the blockade of NLRP3. Moreover, the deubiquitinating enzyme BRCC3 was shown to directly deubiquitinate NLRP3, thus protecting it from proteasomal degradation.^138^ Another layer of NLRP3 regulation is its phosphorylation on Ser291 (or Ser295 in humans) by protein kinase A, which induces its K48- and K63-linked ubiquitination and subsequent proteasomal degradation.^139,140^ Interestingly, Golgi-specific protein kinase D was proposed to phosphorylate NLRP3 on the same serine residue and instead promote NLRP3 activation.^141^ This dichotomy underlines the multidimensional regulation of NLRP3, which depends not only on PTMs but also on its cellular localization. Other phosphorylation/dephosphorylation events control NLRP3 activity after priming. Song et al. showed that after LPS priming, NLRP3 is phosphorylated on Ser194 by c-Jun N-terminal kinase (JNK)1, which facilitates the oligomerization of NLRP3 upon activation by canonical stimuli.^142^ Another study showed that in monocytic cells, dephosphorylation of NLRP3 on Tyr861 by protein tyrosine phosphatase nonreceptor 22 (PTP22) induced NLRP3 activation.^143^ We showed that dephosphorylation of NLRP3 on Ser5 by phosphatase 2A is a prerequisite for its interaction with ASC and downstream activation of caspase-1 in macrophages.^144^ Whether these pathways synergize to tightly control NLRP3 activity remains elusive. It is somewhat more likely that these pathways are engaged in a cell type- and stimulus-specific manner, an explanation that would correspond better to the requirement for different upstream effectors and different subcellular localizations of NLRP3.

The regulation of NLRP3 function is the best-studied example of the tight regulation involved in the activity of inflammasome sensors and the downstream induction of pyroptosis. Other sensors, such as NLRP1 and NLRC4, are also regulated through a combination of ubiquitination and phosphorylation or require cleavage for their activation.^145^ Remarkably, much progress has recently been made in the understanding of NLRP1 activation and the identity of the NLRP1 ligand. In epithelial cells, both mouse NLRP1B and human NLRP1 require cleavage of their C-terminal CARD by pathogen-derived proteases to activate caspase-1.^146–148^ While the existence of a potential ligand for human NLRP1 has long remained elusive, Bauernfried et al. have shown that NLRP1 senses double-stranded RNA molecules such as those generated by Semliki Forest virus replication.^149^ However, whether binding to dsRNA and cleavage of the NLRP1 C-terminal domain are interdependent remains to be elucidated. Interestingly, CARD8, another inflammasome receptor related to NLRP1 (also containing a C-terminal FIIND-CARD structure), was recently discovered. Normally repressed by dipeptidyl peptidase (DPP) 8 and 9, CARD8 is activated in CD4^+^ and CD8^+^ T cells treated with DPP8/9 inhibitors and induces an ASC-independent form of pyroptosis.^150,151^ CARD8 activation requires an autoprocessing step with subsequent degradation of its N-terminal region by the proteasome to free the C-terminal FIIND-CARD region, allowing direct recruitment of caspase-1.^152^

The evolution of such complex regulatory mechanisms across all effector arms of pyroptosis is a clear testimony to the harmful effects of this form of cell death. Remarkably, the discovery of GSDMD rapidly commenced a race by many pharmaceutical companies to develop GSDMD inhibitors, which could constitute flexible options for treating a plethora of pyroptosis-related chronic inflammatory diseases. Time will tell whether one-shot blockade of all propyroptotic pathways is a viable clinical option.

## Breaking the silence of apoptosis

Apoptosis is a mechanism of regulated cell death conserved across the animal kingdom that has been the subject of intensive research for the past three decades and was, for most of this period, believed to be the only regulated type of cell death. An essential feature of apoptosis is the release of cytochrome c from mitochondria, regulated by a balance between proapoptotic and antiapoptotic proteins of the BCL-2 family, initiator caspases (caspase-8, -9 and -10) and effector caspases (caspase-3, -6 and -7). Apoptosis culminates in the breakdown of the nuclear membrane by caspase-6, the cleavage of many intracellular proteins (e.g., PARP and lamin), membrane blebbing, and the breakdown of genomic DNA into nucleosomal structures.^153,154^ These events are hallmarks of apoptosis and are commonly used to identify the cell death pathway engaged (e.g., DNA laddering and caspase-3 and PARP cleavage).

### Apoptotic pathways and regulation

**Fig. 3:**
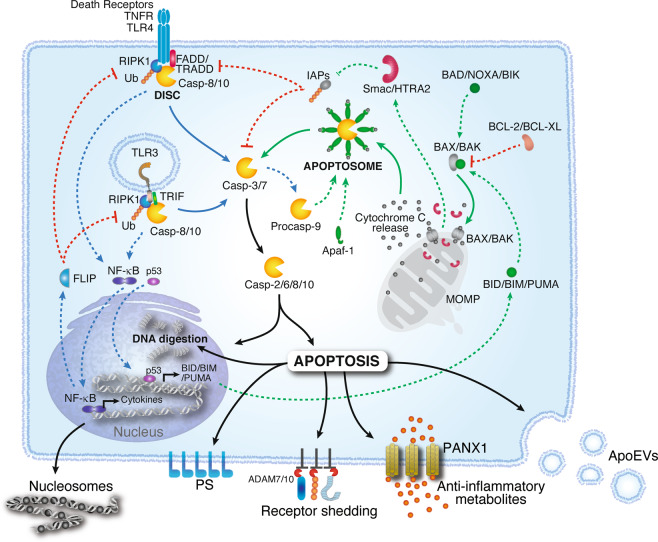
Apoptosis is triggered through two major pathways referred to as the intrinsic and extrinsic pathways. The intrinsic pathway is activated by oligomerization of the B-cell lymphoma-2 (BCL-2) family proteins BAK and BAX. BAK/BAX oligomers form pores in the mitochondrial outer membrane, leading to the release of cytochrome c into the cytosol. Activation of BAK/BAX is regulated by proapoptotic (e.g., BAD and BID) or antiapoptotic (e.g., BCL-2) BCL-2 family proteins. Cytochrome c binds to Apaf-1, which recruits procaspase-9, forming the apoptosome. In the apoptosome, caspase-9 is activated by autoproteolytic cleavage, initiating the caspase-processing cascade. The extrinsic pathway is activated by engagement of membrane receptors such as Tumor necrosis factor (TNF) receptor 1 (TNFR1), death receptors, or Toll-Like Receptors (TLRs). These proteins induce the formation of signaling complexes involving TNFR1-associated death domain protein (TRADD) or Fas-associated death domain protein (FADD), receptor-interacting serine/threonine protein kinase 1 (RIPK1) and procaspase-8. Ubiquitylation of RIPK1 by cellular inhibitors of apoptosis (cIAPs) stabilizes the complex and induces the activation of the transcription factor NFκB. FLIP, also present in the DISC, limits caspase-8 activity while promoting cell survival, cell proliferation, and the production of proinflammatory cytokines. Imbalances in this pathway, such as those imposed by cellular stress, allow the activation of caspase-8 and caspase-10, which in turn triggers the caspase activation cascade. Once active, executioner caspases (i.e., caspase-2, -6, -8 and -10) bring about programmed apoptotic death. Apoptotic cells release messengers in the form of nucleosomal structures, shed receptors, anti-inflammatory metabolites or molecules packaged in apoptotic extracellular vesicles (ApoEVs). Phosphatidylserine (PS) molecules exposed on the outer surface of the plasma membrane function as “eat me” signals for phagocytes

Mechanistically, two main pathways contribute to the caspase activation cascade downstream of mitochondrial cytochrome c release: the *intrinsic* and *extrinsic* pathways (Fig. 3).

The intrinsic pathway is triggered by dysregulation of or imbalance in intracellular homeostasis by toxic agents or DNA damage. It is characterized by mitochondrial outer membrane permeabilization (MOMP), which results in the release of cytochrome c into the cytosol. MOMP and the release of cytochrome c trigger the formation of apoptosomes and caspase-3 activation^155^ and are generally considered the point of no return in apoptotic cell death. The release of cytochrome c to the cytosol is promoted by proapoptotic proteins of the BCL-2 family, such as BAX, BAK, and PUMA. This family can be further divided into activators (e.g., BIM, BID, and PUMA)^156–158^ and sensitizers (e.g., BAD, NOXA, and BIK).^159^ Activator proteins bind directly to apoptosis’s main effectors (BAX and BAK), which undergo conformational changes allowing their oligomerization.^156–161^ Oligomerized BAX and BAK form pores in the mitochondrial membrane, causing MOMP.^162,163^ Sensitizer proteins contribute to apoptosis by inhibiting antiapoptotic factors or controlling the cellular localization of BAX and BAK monomers.^161,164^ Interestingly, other members of the BCL-2 family, BCL-2 and BCL-XL, inhibit apoptosis.^159,165–167^ These antiapoptotic proteins possess two BCL-2 homology (BH3) domains, which form a binding groove that sequesters activator or sensitizer BCL-2 proteins or the BAX and BAK complexes.^158,168^ A fine balance of binding affinities and expression levels of BCL-2 proteins ultimately regulates sensitivity or resistance to apoptosis. This balance is further influenced by posttranslational modification^169,170^ and the cytoplasmic localization^171^ of BCL-2 proteins. Furthermore, cyclin-dependent kinases moderate apoptosis by transcriptionally repressing sensitizers^158,172,173^ or stabilizing antiapoptotic factors such as Mcl-1.^174–176^ The transcription factor p53, one of the first responders to DNA damage and a trigger of the DNA repair machinery and cell cycle arrest, is another proapoptotic regulator. P53 targets promoter regions that control the expression of several proapoptotic BCL-2 proteins.^177–179^ These data emphasize the delicate balance of apoptotic processes, with both the pro- and antiapoptotic teams pulling hard on the rope in a “tug-of-death” game.

MOMP also induces the formation of the apoptosome, a massive complex comprising cytochrome c, apoptosis protease-activator factor 1 (Apaf-1), dATP, and procaspase-9.^180–184^ The assembled apoptosome activates the apoptosis initiator caspase-9. Caspase-9, in turn, cleaves the proforms of the proteases caspase-3 and caspase-7 and unleashes their apoptotic executioner function.^182,183,185–187^ Interestingly, similar to their roles in inhibiting RIPK1/RIPK3-dependent necroptosis, proteins of the IAP family (IAP1/2 and XIAP) can also inhibit caspase-3 activation.^187–191^ Furthermore, XIAP interacts with caspase-9 and caspase-7, thus retaining tight control of the apoptotic process.^187,189,191–193^ However, Smac and HTRA2, released with cytochrome c during MOMP, function as inhibitors of XIAP,^188,192,194–200^ promoting apoptosis. Caspase-3 itself can further promote apoptosis by processing caspase-9 into a p10 fragment lacking the XIAP docking domain and thus evading inhibition.^185^ Once activated, as the last step of the apoptotic process, caspase-3 and caspase-7 cleave several other procaspases (i.e., caspase-2, -6, -8, and -10) into their active forms, creating an apoptosis-amplifying cascade.^153,154,185,201,202^

The second arm of the apoptotic pathway, known as the extrinsic pathway, is initiated by activation of cell surface death receptors. Proapoptotic death receptors include TNFR1/2,^203,204^ Fas,^205,206^ and the TNF-related apoptosis-inducing ligand (TRAIL) receptors DR4^190,207–209^ and DR5.^190,208–212^ Upon activation by their ligands (i.e., TNFα, FasL or TRAIL, respectively), death receptors oligomerize to form platforms at the cell surface. This event leads to the recruitment of adapter proteins (TRADD and FADD) and the activation of the apoptotic initiator caspases caspase-8 and -10, forming the death-inducing signaling complex (DISC).^213–219^ Activation of caspase-8 and -10 is regulated by a caspase-like protein, FLIP, also present in the DISC.^220,221^ Another protein regulating the activation of the extrinsic apoptotic pathway is RIPK1. Indeed, even before necroptosis was discovered, RIPK1 had long been known to be recruited to the DISC, interacting with FADD and TRADD through binding with their death domains.^214,222,223^ Through posttranslational modifications, RIPK1 can promote both a prosurvival NFκB-mediated pathway and a prodeath pathway either through apoptosis or, in the absence of active caspase-8, through necroptosis, as described earlier.^42,224^ At early stages of activation, recruitment of TRADD, procaspase-8, ubiquitinated RIPK1, and the regulatory protein FLIP to the DISC limits the proapoptotic function of caspase-8, while promoting the proinflammatory and NFκB-dependent signaling pathway.^214,225,226^ This prosurvival complex, also known as complex I, is later either internalized or completely detached from the receptor to promote the formation of complex II, which has proapoptotic activity.^214,222^ The importance of the integrity of complex I and RIPK1 ubiquitylation prior to the induction of apoptosis was recently underscored in a study in which targeting TRADD with specific small molecule inhibitors blocked apoptosis and activated the beclin-1-mediated autophagic pathway.^227^ Within complex II, procaspase-8 and procaspase-10 are activated through autocatalytic cleavage.^217,228,229^ This cleavage, in turn, activates effector caspases (i.e., caspase-3, -6 and -7) either directly through proteolytic cleavage^229–231^ or indirectly by activating the BCL-2 protein family member BID, thus generating feedback into the intrinsic pathway and promoting MOMP.^232–236^

### Apoptosis is anti-inflammatory but not silent

The existence of “*Find me*” or “*Eat me*” signals released during apoptosis to recruit phagocytic cells and promote cell clearance has been known for some time.^237^ However, the full regulatory effect of the released contents of apoptotic cells (i.e., secretome) is only beginning to be understood. Recently, Tanzer et al. found a specific secretome signature that distinguishes apoptosis from other forms of cell death. As part of this signature, apoptotic cells mainly release nucleosome components.^72^ Interestingly, this group also detected ectodomain portions of cell surface proteins shed by metalloproteinases (ADAM10/17), most of which were common to both necroptosis and apoptosis. Shedding of cell surface proteins enables dying cells to detach from their sites in the tissue and block signal transduction in dying cells. Furthermore, receptor shedding can have an anti-inflammatory effect through the generation of soluble decoy receptors. In line with the release of active anti-inflammatory signaling molecules from apoptotic cells, apoptotic extracellular vesicles (ApoEVs) have been shown to perform immunoregulatory functions. Indeed, during apoptosis, phosphatidylserine is actively externalized via a caspase-3/7-dependent mechanism activating the scramblase Xkr8.^238,239^ Once at the surface of ApoEVs, PS acts as an “eat me” signal. Notably, cancer cells have the ability to suppress the expression of Xkr8, a mechanism by which they evade detection by phagocytes.^238^ Furthermore, PS promotes the expression and secretion of the anti-inflammatory factor TGF-β by phagocytes, thus underlying its local and systemic immunosuppressive function.^240,241^ Further supporting a role for ApoEVs in shaping the immune response of surrounding cells, ApoEVs have been studied for their capacity to aid antigen presentation. Indeed, ApoEVs released from *Mycobacterium tuberculosis*-infected cells are engulfed by antigen-presenting cells and prime naive CD4^+^ and CD8^+^ T cells toward antimicrobial immunity.^242–244^

Recently, Medina et al. discovered that apoptotic lymphocytes and macrophages release specific anti-inflammatory metabolites while preserving their plasma membrane integrity.^245^ Importantly, this group showed that specific metabolic pathways remain active in apoptosis-committed cells and that the release of metabolites is a dynamic process regulated by caspase-1-dependent opening of pannexin-1 (PANX1) channels. Furthermore, this group found that these metabolites induce gene programs in neighboring cells after release, promoting suppression of inflammation, cell proliferation, and wound healing. Hence, apoptotic cells not only promote their own phagocytosis (i.e., through “find me” or “eat me” signals) but also actively dampen inflammation and promote tissue repair in their surroundings.

### Apoptosis in health and disease

Apoptosis is an indispensable physiological mechanism of tissue formation during embryogenesis and even after birth, when constant cellular turnover and tissue regeneration are particularly essential. Indeed, essential regulatory mechanisms that depend on RIPK1 and caspase-8 orchestrate the fate of embryonic cells during development.^50–52^ Apoptosis is also a necessary tool of the immune system for fighting infections and removing cells with irreparable DNA damage. For example, apoptosis is induced by release of granzyme family molecules from activated T cells and NK cells. Indeed, these proteolytic enzymes were found to target several caspases^219,246^ or BID^247–249^ to convert these mediators into their active form, thus bypassing upstream signals to force targeted cells toward apoptosis. Interestingly, BID is the main target of intracellular proteases upon exposure to cellular stress. Indeed, cathepsins, proteases generally restricted to the lysosomal compartment, are released into the cytosol after lysosomal damage, where they target BID.^250,251^ Similarly, calpain proteases, which have little activity at neutral pH and require high cytosolic calcium levels, were also shown to activate BID following ischemic reperfusion injury or cisplatin treatment.^252,253^ Because of their direct effect on apoptosis initiation, these proapoptotic pathways have been utilized in several therapeutic strategies for cancer.^251,253^ Furthermore, several antiapoptotic proteins (e.g., BCL-2, BCL-XL, and IAPs) are upregulated in cancer cells, thus inhibiting apoptosis and promoting tumor survival. Hence, Smac mimetics have been developed as endogenous proapoptotic therapies in cancer.^43^ Similarly, many other antiapoptotic factors are targeted by anticancer drugs using recombinant domain mimetics as decoys (e.g., BH3), engineered peptides as inhibitors of protein–protein interactions (e.g., stapled peptides), or small molecule drugs targeting a large variety of factors to either activate them (e.g., the BAX agonists SMBA1/3) or block them (e.g., the MCL-1 inhibitor AM-8621).^254^ Notably, p53 induces the expression of both proapoptotic BCL-2 family members and specific death receptors (i.e., Fas and DR5).^177,210,255^ Therefore, therapeutic strategies promoting p53 activity (e.g., cisplatin^256^) may facilitate apoptosis induction through both the intrinsic and extrinsic pathways. Novel anticancer therapeutic strategies targeting caspases with small molecule drugs or by gene editing in solid tumors are under development.^257^

In the context of infection, viruses have evolved a mechanism to modulate apoptosis by mimicking the host’s regulatory systems. Examples include human herpesvirus-8 and molluscipoxvirus, which express FLIP-like inhibitory proteins, coined v-FLIPs, that mimic host proteins and inhibit proapoptotic signaling by death receptors.^258,259^ Similarly to necroptosis, cytomegalovirus (CMV) has evolved mechanisms to counter apoptotic pathways. Human CMV expresses vMIA, which inhibits BAX by binding and sequestering it at the mitochondrial membrane.^260,261^ Subsequent studies also found that vMIA can interact with and inhibit BAK.^262,263^ Unlike human CMV, mouse CMV expresses two proteins, each possessing either BAX- or BAK-inhibitory properties. Mouse CMV-expressed vMIA interacts and blocks BAX,^263–266^ while vIBO prevents BAK oligomerization.^267^ Hence, CMV has mastered strategies to evade two critical cell death pathways by generating inhibitors of crucial components of apoptosis and necroptosis. Although human CMV is known to activate the AIM2 inflammasome,^268^ no evidence that CMV directly prevents pyroptosis, except for its ability to partially downregulate NLRP3 priming in vitro, has been reported to date.^269^

## Crossing lines of regulated cell death

Cell death pathways have long been considered to function in parallel with little or no overlap. However, it is currently clear that apoptosis, necroptosis, and pyroptosis are tightly connected and can crossregulate each other.

The critical function of caspase-8 as a mediator of the apoptotic and necroptotic pathways was one of the earliest-discovered bridges between different types of cell death. Caspase-8 not only regulates apoptosis but is also a central component of the ripoptosome,^42^ where it represents a crucial switch for the cleavage of RIPK1 and RIPK3^270–272^ and one of the TRAF2- or RIPK1-deubiquitylating enzymes, CYLD.^273^ In this way, caspase-8 prevents the formation of necrosomes and favors apoptosis over necroptosis. FADD-mediated activation of caspase-8 downstream of death receptor activation triggers apoptosis,^274–276^ while the absence or pharmacological blockade of caspase-8 drives necroptosis.^277^ Interestingly, while caspase-8 seems to play a role in the stabilization of the ripoptosome, its proteolytic activity is required to prevent necroptosis.^278^ Hence, caspase-8 in the DISC and the ripoptosome is critical for both apoptosis and necroptosis. Moreover, several studies noted a link between the activation of the ripoptosome and caspase-1 and the release of IL-1β and IL-18 after bacterial infection.^54,55,279^ A study by Gurung et al. placed FADD and caspase-8 upstream of NLRP3 activation, noting that both molecules contribute to macrophage priming and activation of caspase-1 and IL-1β maturation.^279^ The same group then went on to show that influenza A virus (IAV) induces RIPK3/caspase-8-dependent cell death through activation of DAI followed by activation of NLRP3.^280^ It was then unclear whether the contributions of RIPK3 and caspase-8 to NLRP3 activation were mediated only by a priming effect. Indeed, the potential autocrine contribution of TNFα or IFN-I signaling induced downstream of TLR4 or DAI could partially explain these findings. Several studies have demonstrated that RIPK3 regulates caspase-8 activity and the subsequent activation of NLRP3 downstream of TLR or TNFR1/2.^92,281–284^ These studies showed that in the absence of RIPK3-ubiquitinating IAPs, stimulation with dsDNA, LPS, or TNFα triggers RIPK3 to activate caspase-8 in myeloid cells. This event promotes apoptosis and NLRP3-dependent caspase-1 activation and IL-1β release. However, in the presence of functional caspase-8, NLRP3 activation and IL-1β release did not require RIPK3 kinase activity or MLKL activation. More recent data show that under these conditions, caspase-8 promotes NLRP3 activation by cleaving GSDMD directly, thereby promoting pyroptosis downstream of TNF and providing protection against infection (e.g., *Yersinia* infection) in vivo.^285–287^ In the absence of caspase-8, RIPK3 kinase activity and MLKL are necessary. However, it is still unclear how MLKL promotes NLRP3 activation in this context. Since active MLKL induces loss of membrane integrity, similar to GSDMD, MLKL-dependent K^+^ efflux may cause NLRP3 activation. Whether NLRP3 activation contributes to cell death via GSDMD under these conditions remains to be explored. Strikingly, caspase-8 activation downstream of the intrinsic apoptosis pathway (i.e., BAX/BAK-dependent mitochondrial destabilization followed by caspase-3/7 activation) has recently been connected to NLRP3 activation and the secretion of bioactive IL-1β from macrophages.^288^ Indeed, activation of BAX/BAK triggers proteasomal degradation of the ubiquitin ligase IAP, allowing caspase-8 activation by caspase-3/-7. Interestingly, although some researchers had already identified GSDMD and GSDME activation downstream of caspase-8 after *Yersinia* infection,^285–287^ in the context of mitochondrial apoptosis, NLRP3 activation induced by K^+^ efflux seemed to be independent of GSDMD. Instead, apoptotic caspases (i.e., caspase-3/-7) inactivate GSDMD through cleavage at aspartate 88.^285,289^ Hence, the exact mechanism by which intrinsic apoptosis triggers NLRP3 activation remains unclear. The last piece of the puzzle was placed with the recent discovery that in macrophages, pannexin-1 (PANX1) channels induced during apoptosis drive NLRP3 activation.^290,291^ Indeed, downstream of intrinsic and extrinsic apoptosis, RIPK3-dependent activation of caspase-3 and subsequent maturation of PANX1 trigger K^+^ efflux, which in turn activates NLRP3 and the release of IL-1β independent of GSDMD or GSDME. However, PANX1 channels are dispensable for canonical and noncanonical inflammasome activation (in which GSDMD is critical). Another link between pyroptosis and apoptosis was recently identified by Tsuchiya et al.^292^ In macrophages, in the absence of GSDMD, activation of caspase-1 redirects cell fate toward caspase-3-, caspase-9- and BID-dependent apoptosis. This finding could explain the only partial reduction in Gsdmd^−/−^ macrophage death described by Kayagaki et al.^120^ How caspase-1 triggers the activation of caspase-3 and further apoptosis remains to be clarified.

As another example of an immune response connecting different cell death pathways, RNA viruses induce NLRP3 activation and the subsequent release of IL-1β in a RIPK1/RIPK3-dependent and MLKL-independent manner.^293^ Viral infection triggers RIPK1/RIPK3-mediated phosphorylation of DRP1, resulting in mitochondrial damage and NLRP3 activation, presumably through the release of mitochondrial ROS. In light of the new data discussed here, RNA virus-induced NLRP3 activation could be the result of caspase-8 engagement downstream of mitochondrial destabilization and the subsequent activation of caspase-3/-7. This hypothesis is worth investigating. Other earlier studies showed that NLRP3 is activated by intracellular detection of bacterial or viral RNA and by synthetic nucleoside mimetics.^100,101^ Whether the RIPK1/RIPK3/MLKL pathway was involved in these experiments remains to be evaluated, taking into consideration a potential role for IFN-I signaling induction.

Further blurring the lines between cell death pathways, TAK1, also found in complex with RIPK1, FADD, and caspase-8 downstream of TNFR or TLR activation, has recently emerged as another prosurvival regulator of cell death. In macrophages, TAK1 deletion was shown to induce caspase-8-dependent cleavage of GSDMD after stimulation with TNFα or TLR ligands.^294^ Furthermore, a new concept has emerged where several players in different cell death pathways merge into a single complex called the PANoptosome. The first evidence of this oligomeric structure came from the work of the Kanneganti laboratory.^295,296^ This group first reported that deficiency or blockade of TAK1 induces NLRP3/RIPK1-dependent cell death. Interestingly, TAK1 blockade triggers proinflammatory pathways reminiscent of those induced by the priming step necessary for NLRP3 activation.^295^ Treatment of cells with TLR ligands markedly abolishes the requirement for RIPK1 kinase activity prior to activation of caspase-1 and other apoptotic or necroptotic caspases (e.g., caspase-3 and caspase-8).^296^ Expression of a kinase-dead version of RIPK1 was necessary and sufficient for activation of caspase-1 and cell death after 12 h of stimulation. Kinetic analysis revealed activation of MLKL in a RIPK3/caspase-8-dependent manner as early as 2 h after TLR or TNFR stimulation. In the context of previous studies by Lawlor et al.,^92,283,288^ early induction of apoptosis or necroptosis could explain the downstream activation of NLRP3/caspase-1 and the induction of pyroptosis. Further work from the same group proposed that several microbes can induce cell death via PANoptosis.^297^ Deletion of several important regulators of cell death pathways has shown that *Salmonella* preferentially induces cell death through pyroptosis, while IAV triggers cell death through RIPK3/caspase-8-dependent necroptosis. Listeria and VSV seem to induce cell death equally through pyroptosis and necroptosis, as only complete knockdown of caspase-1/11, RIPK3 and caspase-8 rescues cell survival. New data also implicate caspase-6 in the activation of DAI/RIPK3/MLKL followed by NLRP3/caspase-1 in macrophages infected with IAV.^298^ It is still unclear whether all pathways are triggered in parallel inside infected cells under these conditions or whether released cytokines and other mediators released from infected cells trigger a different cell death pathway in bystander cells. Analysis of the different cell death pathways engaged on a single-cell level could help deconvolute this issue.

Supporting the role of caspase-8 in pyroptosis, new data link caspase-8 to the formation of ASC specks in embryonic intestinal tissue.^299^ Using a combination of transgenic mice with deletion of cell death regulators, Newton and colleagues demonstrated that inactivation of procaspase-8 (i.e., through the loss-of-function C362A mutation) seeds the formation of ASC specks, thus promoting caspase-1-dependent cleavage of GSDMD and pyroptosis.

A recent study by Gitlin et al. added another key to caspase-8’s toolbox.^300^ In macrophages, downstream of TRIF-dependent TLRs (i.e., TLR3 and TLR4), activated caspase-8 cleaves the IFN-induced suppressor of cytokine production N4BP1, amplifying cytokine production and release. MyD88-dependent TLRs fail to target N4BP1, resulting in a reduced cytokine response. However, engagement of TNFR1 by TNFα followed by caspase-8 activation and N4BP1 cleavage allows TRIF-independent TLRs to induce cytokine production. Since coactivation of TLRs and TNFR can likely occur during pathogen infection in vivo, engagement of TNFR induces cleavage of N4BP1 by caspase-8 to allow TRIF-independent TLRs to enhance cytokine responses. Hence, caspase-8 is a critical node in signal integration by which TNF permits cytokine and chemokine production downstream of TRIF-independent TLRs.

**Fig. 4:**
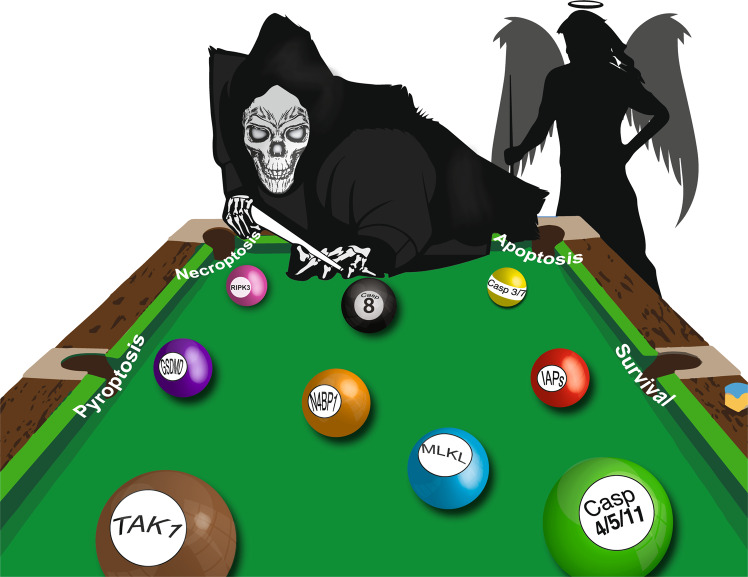
Crossing lines of programmed cell death. The different cell fates are balanced by an intricate game between pro- and antisurvival factors in which caspase-8 seems to be the central referee

Hence, caspase-8 is a central regulator of cell death, acting as a cellular compass to promote apoptosis, necroptosis, or pyroptosis depending on its posttranslational state and the cell type (Fig. 4). Regulators other than caspase-8 have emerged as gatekeepers between cell death pathways. For example, GBP1 was recently proposed to act as a microbe-specific switch enabling recognition of either *Toxoplasma gondii* DNA, inducing apoptosis through AIM2, or *Salmonella* LPS, instead promoting pyroptosis via caspase-4.^301,302^

Collectively, these data emphasize the tight crossregulation that exists between apoptosis, necroptosis, and pyroptosis and indicate the presence of bridges between the pathways to coordinate cell death should one pathway become compromised. These data also highlight that while apoptosis is less immunogenic than necroptosis or pyroptosis, it enables the release of proinflammatory signaling molecules such as IL-1β under specific conditions. Hence, cell death pathways are not independent entities acting in parallel. Instead, they must be considered flexible molecular tools generating a wide range of outcomes, emitting signals with a full spectrum of anti- or proinflammatory components. Rather than a punctual event, the nature of the “death signal” is likely to result from the tipping of a delicate balance between pro- and antideath signals. At that point, the state of the cell, especially the status of switch regulators such as caspase-8, determines the pathway to be executed.

## Conclusion/perspectives

As summarized herein, many important discoveries have recently been made in the field of cell death. Several new players have been identified that often underline new connections bridging distinct cell death pathways. Cell death, as it turns out, is a very intricate game where distinct central players have the power to tip a fragile balance from life to death and from pro- to anti-inflammatory signals for the cell environment. The next years will certainly be exciting in the field as more light is shed on the complex regulatory mechanism that governs cell death. These years will also be a time during which new drugs are developed, enabling these discoveries to be utilized to either enhance (e.g., cancer treatments) or prevent (e.g., treatment of inflammatory diseases) cell death.
